# Supplementary material for: Chemistry and Functions of Imported Fire Ant Venom
Source: Toxins (Basel). 2023 Aug 3;15(8):489. doi: 10.3390/toxins15080489 (PMC10467070; doi:10.3390/toxins15080489)
Supplement: Supplementary file 1 [file toxins-15-00489-s001.zip › toxins-2470522-supplementary.docx]

| Chemistry and Functions of Imported Fire Ant Venom  **Supplementary Materials**  **Jian Chen**  **Table S1.** Venom alkaloids in *Solenopsis* ants excluding *S. invicta* and *S. richteri* | | | |
| --- | --- | --- | --- |
| **Species** | **Compounds** | **Reference** | |
| *Solenopsis carolinensis* Forel | *trans*-2me-6-nonylpiperidine | [1] | |
| *Solenopsis conjurata* Wheeler | 3-hexyl-5me-indolizine | [2] | |
|  | 3-ethyl-5me-indolizine | [2] | |
|  | *cis*-isosolenopsin A | [2] | |
|  | *trans*-2me-6-nonylpiperidine | [2] | |
|  | *cis*-2me-6-nonylpiperidine | [2] | |
| *Solenopsis fugax* Latreille | *trans*-2-butyl-5-heptylpyrrolidine | [3] | |
| *Solenopsis geminata* Fabricius | *cis*-isosolenopsin A | [4,5] | |
|  | *trans*-solenopsin A | [5] | |
|  | *cis*-isosolenopsin B | [6] | |
|  | *cis*-2me-6-Z4-tridecenyl-piperidine | [6] | |
| *Solenopsis geminata rufa* Jerdon | *cis*-isosolenopsin A | [7] | |
|  | *trans*-solenopsin A | [7] | |
| *Solenopsis globularia pacifica* Wheeler | *trans*-2me-6-nonylpiperidine | [7] | |
|  | *cis*-2me-6-nonylpiperidine | [7] | |
| *Solenopsis littoralis* Smith | *trans*-solenopsin B | [1] | |
| *Solenopsis maboya* Snelling | *trans*-2me-6-nonylpiperidine | [8] | |
|  | *cis*-2-heptyl-5me-piperidine | [8] | |
| *Solenopsis maniosa* Wheeler | *cis*-isosolenopsin A | [7] | |
|  | *trans*-solenopsin A | [7] | |
| *Solenopsis molesta* Say | *E*5*E*9-3-hexyl-5-methylindolizidine | [9] | |
|  | *Z*5*E*9-3-hexyl-5-methylindolizidine | [9] | |
|  | *cis*-2me-6-nonylpiperidine | [9] | |
| *Solenopsis pergandei* Forel | *trans*-solenopsin A | [1] | |
| *Solenopsis punctaticeps* Mayr | *trans*-2-butyl-5-heptylpyrrolidine | [10] | |
| *Solenopsis saevissima* Smith | *cis*-isosolenopsin A | [6,11] | |
|  | *trans*-solenopsin A | [6,11] | |
|  | *cis*-isosolenopsin B | [6,11] | |
|  | *cis*-2me-6-Z4-tridecenyl-piperidine | [6,11] | |
|  | *trans*-solenopsin B | [6,11] | |
|  | *trans*-2me-6-Z4-tridecenyl-piperidine | [6,11] | |
|  | 2me-6-undecenylpiperidine | [6,11] | |
| *Solenopsis* sp | *trans*-2me-6-nonylpiperidine | | [12] |
|  | *cis-*2me-6-nonylpiperidine | | [12] |
|  | 2me-6-*Z*4-nonylpiperidine | | [12] |
|  | *Z*5Z*9*-3-hexyl-5-methylindolizidine | | [12] |
|  | *Z*5Z*9*-3-butyl-5-propylindolizidine | | [12] |
|  | *E*5*E*9-3-butyl-5-propylindolizidine | | [12] |
|  | 2me-6-nonylpiperidine | | [1] |
| *Solenopsis steinheilli* Forel | *trans*-2me-6-nonylpiperidine | | [7] |
|  | *cis*-2me-6-nonylpiperidine | | [7] |
|  | *N*-methyl-2me-6-nonylpiperidine | | [7] |
| *Solenopsis tennesseensis* Smith | *Z*5*E*8-3-heptyl-5me-pyrrolizidine | | [13] |
| *Solenopsis xyloni* Buren | *cis*-isosolenopsin A | | [5,6] |
|  | *trans*-solenopsin A | | [5,6] |
|  | *cis*-isosolenopsin B | | [6] |
|  | *cis*-2me-6-Z4-tridecenyl-piperidine | [6] | |


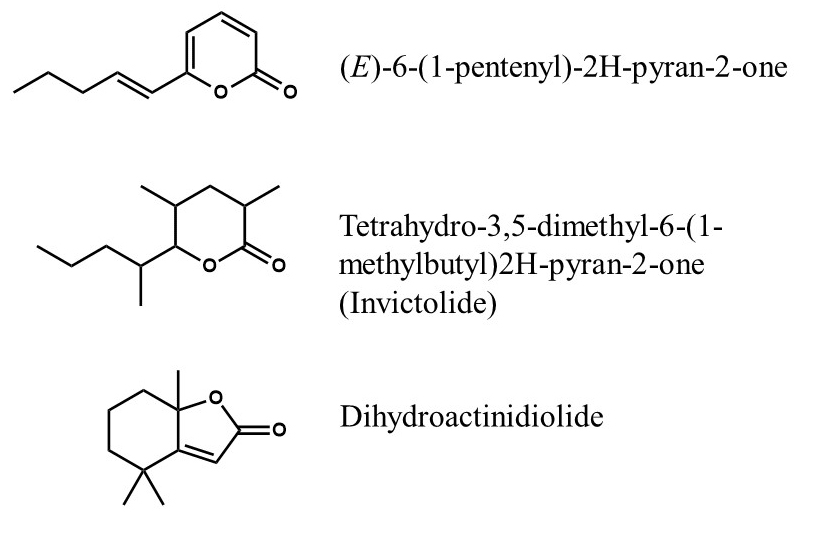


**Figure S1.** Chemical structures of *Solenopsis* *invicta* queen recognition pheromone components. These compounds were determined by Rocca et al. in 1983[14,15].

References

1. Jones, T.H.; Blum, M.S.; Fales, H.M. Ant venom alkaloids from Solenopsis and Monomorium species. Recent developments. *Tetrahedron* **1982**, *38(13)*, 1949-1958, doi: doi:10.1016/0040-4020(82)80044-6
2. Jones, T.H.; Highet, R.J.; Blum, M.S.; Fales, H.M. (5Z,9Z)-3-alkyl-5-methylindolizidines from Solenopsis (Diplorhoptrum) species. *Journal of Chemical Ecology* **1984**, *10(8)*, 1233-1249, doi: doi:10.1007/BF00988551
3. Blum, M.S.; Jones, T.H.; Hölldobler, B.; Fales, H.M.; Jaouni, T. Alkaloidal venom mace: offensive use by a thief ant. *Naturwissenschaften* **1980**, *67(3)*, 144-145, doi: http://dx.doi.org/10.1007/BF01073620
4. Cruz-López, L.; Rojas, J.C.; Cruz-Cordero, R.d.l.; Morgan, E.D. Behavioral and chemical analysis of venom gland secretion of queens of the ant Solenopsis geminata. *Journal of Chemical Ecology* **2001**, *27(12)*, 2437-2445, doi: doi:10.1023/A:1013671330253
5. Brand, J.M.; Blum, M.S.; Ross, H.H. Biochemical evolution in fire ant venoms. *Insect Biochemistry* **1973**, *3(9)*, 45-51, doi: doi:10.1016/0020-1790(73)90017-6
6. Brand, J.M.; Blum, M.S.; Fales, H.M.; MacConnell, J.G. Fire ant venoms: Comparative analyses of alkaloidal components. *Toxicon* **1972**, *10(3)*, 259-271, doi: doi:10.1016/0041-0101(72)90011-6
7. Blum, M.S.; Jones, T.H.; Lloyd, H.A.; Fales, H.M.; Snelling, R.R.; Lubin, Y.; Torres, J. Poison gland products of Solenopsis and Monomorium species. *Journal of Entomological Science* **1985**, *20*, 254-257.
8. Torres, J.A.; Zottig, V.E.; Co, J.E.; Jones, T.H.; Snelling, R.R. Caste specific venom chemistry of Solenopsis maboya and S. torresi (Hymenoptera: Formicidae). *Sociobiology* **2001**, *37*, 579-583.
9. Gorman, J.S.T.; Jones, T.H.; Spande, T.F.; Snelling, R.R.; Torres, J.A.; Garraffo, H.M. 3-hexyl-5-methylindolizidine isomers from thief ants, Solenopsis (Diplorhoptrum) species. *Journal of Chemical Ecology* **1998**, *24(5)*, 933-943, doi: doi:10.1023/A:1022381719471
10. Pedder, D.J.; Fales, H.M.; Jaouni, T.; Blum, M.; MacConnell, J.; Crewe, R.M. Constituents of the venom of a South African fire ant (Solenopsis punctaticeps). 2,5-dialkylpyrrolidines and -pyrrolines, identification and synthesis. *Tetrahedron* **1976**, *32(19)*, 2275-2279, doi: doi:10.1016/0040-4020(76)88001-5
11. Fox, E.G.P.; Pianaro, A.; Solis, D.R.; Delabie, J.H.C.; Vairo, B.C.; MacHado, E.D.A.; Bueno, O.C. Intraspecific and intracolonial variation in the profile of venom alkaloids and cuticular hydrocarbons of the fire ant Solenopsis saevissima smith (Hymenoptera: Formicidae). *Psyche* **2012**, *2012, Article ID 398061*, 10 p., doi: http://dx.doi.org/10.1155/2012/398061
12. Jones, T.H.; Torres, J.A.; Spande, T.F.; Garraffo, H.M.; Blum, M.S.; Snelling, R.R. Chemistry of venom alkaloids in some Solenopsis (Diplorhoptrum) species from Puerto Rico. *Journal of Chemical Ecology* **1996**, *22(7)*, 1221-1236, doi: doi:10.1007/BF02266962
13. Jones, T.H.; Blum, M.S.; Fales, H.M.; Thompson, C.R. (5Z,8E)-3-heptyl-5-methylpyrrolizidine from a thief ant. *Journal of Organic Chemistry* **1980**, *45(23)*, 4778-4780, doi: doi:10.1021/jo01311a048
14. Rocca, J.R.; Tumlinson, J.H.; Glancey, B.M.; Lofgren, C.S. The queen recognition pheromone of Solenopsis invicta, preparation of (E)-6-(1-pentenyl)-2H-pyran-2-one. *Tetrahedron Letters* **1983**, *24(18)*, 1889-1892, doi: doi:10.1016/S0040-4039(00)81798-0
15. Rocca, J.R.; Tumlinson, J.H.; Glancey, B.M.; Lofgren, C.S. Synthesis and stereochemistry of tetrahydro-3,5-dimethyl-6-(1-methylbutyl)-2H-pyran-2-one, a component of the queen recognition pheromone of Solenopsis invicta. *Tetrahedron Letters* **1983**, *24(18)*, 1893-1896, doi: doi:10.1016/S0040-4039(00)81799-2
